# Determinants of birth asphyxia at public hospitals in Ilu Aba Bor zone southwest, Ethiopia: a case control study

**DOI:** 10.1038/s41598-022-15006-y

**Published:** 2022-06-23

**Authors:** Tarekegn Fekede, Abeya Fufa

**Affiliations:** 1 Department of Nursing, College of Health Science, Mettu University, Mettu, Ethiopia; 2 Department of Midwifery, College of Health Science, Metu University, Metu, Ethiopia

**Keywords:** Health care, Medical research

## Abstract

Birth asphyxia is a leading cause of neonatal deaths, which accounts for about 31.6% of all neonatal deaths in Ethiopia. Despite its being one of the important causes of morbidity and mortality in newborns, its determinants were not investigated according to local context. So, this study was aimed at investigating the determinants of asphyxia at Illu Aba Bor zone public health facilities. An institution-based case–control study was employed. A pre-tested, structured and adapted interviewer administered questionnaire for mothers of newborn interviews and a data extraction tool for chart review were used. The collected data were entered into Epi-data version 3.1 and exported to SPSS version 24 for further analysis. A binary logistic regression was employed, and variables with a *p*-value < 0.25 were taken to a multi-variable logistic regression. Finally, a Bonferroni correction was used and variables with a *p*-value < 0.0038 at 95% CI were declared statistically significant. A total of 308 (103 cases vs 205 controls) mothers of newborns were interviewed, yielding a response rate of 100%. The mean age (SD) of mothers for the cases and the controls were (25.97 ± 4.47) and (25.52 ± 4.17) respectively. Prolonged duration of labor [AOR 4.12; 95% CI 1.78, 9.50], non-cephalic fetal presentation [AOR 4.35; 95% CI 1.77, 10.67], being preterm [AOR 5.77; l95% CI 2.62, 12.69] and low birth weight [AOR 4.43; (95% CI 1.94, 10.13) were found to be the determinants of birth asphyxia. Prolonged duration of labor, non-cephalic presentation, prim parous, preterm, and low birth weight were the independent determinants of birth asphyxia. Hence, improving the utilization of parthograph during labor and interventions focusing on this area should give priority to reducing the risk of morbidity and mortality.

## Introduction

Birth asphyxia is the most common and serious neonatal health problem globally, and it significantly contributes to neonatal morbidity^1^. It can be defined as a “failure to initiate and sustain spontaneous breathing at birth^2^”, and it’s diagnosed in extramural babies per minute or intramural babies with a birth Asphyxia-Apgar score of less than 7 in the first 5 min^3^. Globally, in 2017 alone, 5.4 million children died before their fifth birthday and 2.5 million of those children died in their first month of life, of which 23% of all deaths were due to asphyxiation, the fifth leading cause of under-5 mortality^4^.

In addition to this, WHO reports indicate that birth asphyxia is the third leading cause of death in newborns after infection and premature birth, which accounts for about 23% in developing countries, mainly Asia and sub-Saharan Africa, 40% & 34% respectively^5^. For example, in sub-Saharan country, Ethiopia reached its MDG4 child mortality goal. Despite the fact that neonatal mortality remains high, this is mainly contributed by birth asphyxia (31.6%)^6^.

The proportion of birth asphyxia is 2 per 1000 live births in developed countries, and it is more than tenfold higher in low-income countries with limited access to quality maternal and neonatal care^7^. Moreover, the proportion of neonatal deaths is high in Sub-Saharan Africa, including, Ethiopia^8^. In Ethiopia, the neonatal mortality rate decreased from 2005–2016 but, it was slightly increased to 30 per 1000 live births by 2019^9,10^. Even though the prevalence of birth asphyxia in Ethiopia varies from place to place, for instance, previous studies done in Tigray, Gondar, Dilla and Jimma with 22.1%, 13.8%, 32.8%, and 18%, respectively^11–14^, the summary of a pooled umbrella review found to be 22.52%^15^.

Furthermore, out of worldwide neonatal deaths, 24% were due to birth asphyxia with serious neurological sequels^3,16–18^.

Even though a number of contributing factors to birth asphyxia existed, the main factors were thought to be categorized into maternal, fetal, and materno-fetal factors^3^. The putative maternal-related risk factors, maternal socio-demographic is one which includes age of the mother, educational status of the mother, and marital status^3,19–25^. In addition to maternal socio-demographic, the past maternal obstetric-related risk factors like no or less ANC follow up^21,22,25^, being primiparous^26,27^, and child death^26^ were also determinants of birth asphyxia. Moreover, meconium-stained amniotic fluid^28,29^ and other labour complications^30,31^ were also risk factors for birth asphyxia.

Despite the high contribution of birth asphyxia to neonatal death, most of the risk factors were preventable and treatable^5,25^. Along with this, most countries have developed guidelines to treat neonatal asphyxia, including Ethiopia^32^. However, neonatal death is still high, ‘world is failing new-borns’ as quoted by UNICEF^21^ and it’s challenging to achieve sustainable development goals (SDGs) of 12 per 1000 live births by 2030^19^. So, to reduce the impact of birth asphyxia on neonatal morbidity and mortality, more studies are needed in a variety of settings, including Ethiopia. Hence, studies are needed to identify the determinants of childbirth in order to avert the problem. Therefore, this study aims to identify the determinants of birth asphyxiation, especially in the study area.

## Methods

### Study design and setting

A facility-based case–control study was used in the Ilu Abba Bora zone public hospitals. The Ilu Aba Bor zone is bordered to the north by the East Wollega zone, to the south by the nations and nationalities, to the west by Kelem Wollega, to the northwest by the West Wollega zone, and to the east by the East Wollega zone. The zone is more than 600 km^2^ away from the capital of Ethiopia, Addis Ababa. The zone includes approximately a total population of 1,197,156, of whom 58, 7134 are men and 610,022 are women; with an area of 16,555.36 square kilometers, Ilu Abba Bor has a population density of 72.31. The zone has two hospitals; one referral and one general hospital actively provide care for the maternal and newborn babies. Monthly, Mettu Karl Comprehensive Referral Hospital provides 320 delivery services and Darimu District Hospital provides about 280 delivery services.

### Study participants

Newborns diagnosed with birth asphyxia by the physician and those eligible for the study during data collection time (cases) and newborns without birth asphyxia and eligible for study during data collection time (control) by excluding newborns with congenital anomalies and birth defects.

In this study, the subjects were divided into cases and controls.

*Cases (asphyxiated)*: Newborns with an APGAR value of < 7 by the doctor at 5 min.

*Controls (not asphyxiated)*: newborns with an APGAR value of > 7 at 5 min.

### Variables and measurements

*Cases (asphyxiated)*: Newborns with an APGAR score (A = Appearance, P = Pulse, G = Grimace, A = Activity, R = Respiration) value of < 7 by the doctor at first and fifth minute^33–35^.

*Controls (not asphyxiated)*: Newborns with an ‘APGAR’ score value of ≥ 7 at first and fifth minutes.

*Prolonged labor*: Occurs when labor after the latent phase of first stage of labor exceeds 12 h in primigravida or 8 h for multipara mothers.

*Prolonged membrane rupture*: A condition in which a rupture of membrane of amniotic sac and chorion occurs 1 h before the onset of labour.

*Anemia*: maternal hemoglobin level < 11 mg/dl during current pregnancy.

*Hypertension during pregnancy*: the type of hypertension which occurs after 28th weeks of gestation.

### Sampling and sampling technique

Sample size was determined by double population proportion formula, using Epi- info version 7 from factors reviewed by considering 95% CI, 80% power, 1:2 case to control ratio, Odds Ratio = 2.21 which is the ratio of odds of cases among neonates with birth asphyxia to odds of controls among neonates free of birth asphyxia; proportion of controls 22.7% and proportion of cases as 39.3%, preterm, unable to read and write, primiparous and ante partum hemorrhage as a variables and the final sample size was 308(103 cases and 205controls) after adding 5% non-respondent rate(23). For sampling purpose, systematic random sampling was used for all delivered neonates in the study as a sampling frame. By exploiting a monthly delivery report from the hospital and accounting for 15.6% of newborns who are asphyxiated. Every second asphyxiated baby was selected as a case, while every third non-asphyxiated newborn was included as a control.

### Data collection tool and procedure

Primary and secondary (record review) data were used. A pre-tested, structured and adapted questionnaire^11,12,35,36^ was used to collect maternal socio-demographic and antepartum characteristics. A pre-tested checklist (extraction tool) was used to collect intra-partum and fetal related factors for both cases and controls. The questionnaire was first compiled in English and translated into the naïve local language, "Afan Oromo," and then translated back into English by the linguist for consistency. Data were gathered by face-to-face interview and reviewed from medical charts by trained BSc nurses after cases were confirmed by physicians (birth asphyxia) and controls were recorded by identification numbers.

### Data quality and analysis

Data were collected by trained health professionals and cleaned, coded and entered into Epi-data version 3.1 and then transported into SPSS version 24 for analysis. Frequencies and cross tabulations were used to summarize descriptive statistics. The association between birth asphyxia and each covariate was first assessed by bivariate logistic regression to identify a candidate variable for the final model. Variables with *p*-value < 0.25 were taken to multi-variable regression. A backward likelihood ratio with a probability distance of 0.1 was used to develop the model. The collinearity diagnosis has been checked. The quality fit of the final model was checked using the Hosmer–Lemeshow test of the quality check, taking into account the good fit at a *p*-value of 0.05 (0.208), the omnibus likelihood test < 0.05(0.000) and the model classification of the Checked accuracy (77.9%). The odds ratios estimated at 95% CI was used to show the strength of the association. Finally, A Bonferroni correction was used and variables with a *p*-value < 0.0038(threshold for significance was *p* = 0.05/13) used to declare statistical significant.

### Research procedure

This study was carried out in accordance with relevant guidelines.

### Ethical consideration

Letter of ethical clearance was obtained from Institutional Review Board of Institute of Health, Jimma University and letter of cooperation from population and family health department. Informed consent was obtained after the purpose of the study was explained to participants. To ensure confidentiality the participants were informed that their data were coded and no need of writing their name.

## Results

### Socio-demographic characteristics

A total of 308 (103 cases and 205 controls) participants took part in this study, with a response rate of 100%. The mean ages (standard deviation) of the mothers for the cases and controls were 25.97 (SD: 4.47) and 25.52 (± SD: 4.17), respectively. Almost three-quarters (72.5%) of the mothers in the cases and 143 (69.8%) of the controls were married (Table 1).

**Table 1:** Socio demographic characteristics of mothers who delivered atIlu Aba Bor, Public Hospitals, Ethiopia, 2021.

Variable	Category	Cases n = 103 (%)	Controls n = 205 (%)	Total n = 308(%)	*p*-value
Age in years	< 18 years	18 (17.48)	38 (18.54)	56 (18.18)	0.125
	18–29 years	58 (56.31)	87 (42.44)	145 (47.08)	
	≥ 30 years	27 (26.21)	80 (39.02)	107 (34.74)	
Marital status	Married	75 (72.8%)	143 (69.8%)	218 (70.78)	0.730
	Unmarried	28 (27.2%)	62 (30.2%)	90 (29.22)	
Educational status	No formal educated	29 (28.16)	34 (16.59)	63 (20.45)	0.025
	Educated	74 (71.84)	171 (83.41)	245 (79.55)	
Occupation	unemployed	85 (82.52)	158 (77.07)	243 (78.90)	0.230
	Employed	18 (17.48)	47 (22.93)	65 (21.10)	

### Past obstetrics related characteristics

Connected to the characteristics of past obstetrics history, twenty-eight (27.2%) mothers of the cases and ninety-one (44.4%) controls were primiparous, while 13 (12.6%) mothers of the cases and 17(8.3%) controls have had a history of child death. Regarding the frequency of antenatal care follow-up, 84(81.6%) of mothers in the cases and 173(74.4%) of controls had antenatal care follow-up more than four times (Table 2).

**Table 2:** Past obstetrics characteristics of mothers who delivered at Ilu Aba Bor, in Public Hospitals, Ethiopia, 2021.

Variable	Category	Cases n = 103(%)	Controls n = 205(%)	Total N = 308(%)	*p*-value
Number of ANC	≥ 4	84 (81.55)	173 (84.39)	257 (83.44)	0.001
Follow up	< 4	19 (18.45)	32 (15.61)	51 (16.56)	
Parity	Multipara	75 (72.82)	114 (55.61)	189 (61.36)	0.017
	Primipara	28 (27.18)	91 (44.39)	119 (38.64)	
Miscarriage	Yes	15 (14.56)	16 (7.80)	31 (10.06)	0.920
	No	88 (85.44)	189 (92.20)	277 (89.94)	
Child death	Yes	13 (12.62)	17 (8.29)	30 (9.74)	0.620
	No	90 (87.38)	188 (91.71)	278 (90.26)	

### Neonatal and intrapartum related characteristics

From the study participants, 20(19.4%) mothers of the cases and 13(6.4%) controls developed prolonged labor. Regarding weight of new born, 21(20.4%) of the cases and 14(6.8%) of the controls had birth weight less 2500 gm. On the other hand, 62(25.2%) of the cases and 15(7.3%) of the controls had gestational age of less than 37 weeks (preterm). Moreover, 98(95.1%) cases and 201 (98.1%) controls had a singleton baby (Table 3).

**Table 3:** Neonatal and Intra-partum characteristics of mothers who delivered at Ilu Aba Bor, in Public Hospitals, Ethiopia, 2021.

Variable	Category	Cases n = 103(%)	Controls n = 205(%)	Total N = 308(%)	*p*-value
Gestational age	≤ 37 weeks	26 (25.24)	15 (7.32)	41 (13.31)	0.002
	> 37 weeks	77 (74.76)	190 (92.68)	267 (86.69)	
Sex of neonates	Female	37 (35.92)	93 (45.37)	130 (42.21)	0.731
	Male	66 (64.08)	112 (54.63)	178 (57.79)	
Newborn weight in gram	≤ 2500 g	21 (20.38)	14 (6.83)	35 (11.36)	0.000
	> 2500 g	82 (79.62)	191 (93.17)	273 (88.64)	
Duration of labour	Normal	83 (80.58)	192 (93.66)	275 (89.29)	0.015
	Prolonged	20 (19.42)	13 (6.34)	33 (10.71)	
Mode of delivery	SVD*	56 (54.37)	166 (80.98)	222 (72.08)	0.25
	CS**	17 (16.50)	11 (5.37)	28 (9.09)	
	Instrument	30 (29.13)	28 (13.65)	58 (18.83)	
Prolonged rupture of membrane	Yes	10 (9.7%)	11 (5.4%)	21 (6.82)	0.002
	No	93 (90.3%	194 (94.6%)	297 (93.18)	
Fetal presentation	Non-	17 (16.51)	11 (5.37)	28 (9.09)	0.001
	cephalic Cephalic	86 (83.49)	194 (94.63)	280 (90.91)	
Delivery outcome	Singleton	98 (95.15)	201 (98.05)	299 (97.08)	0.310
	Multiple	5 (4.85)	4 (1.95)	9 (2.92)	

*SVD, Spontaneous vaginal delivery; **CS, Cesarean Section.

### Medical related characteristics

**Figure 1:**
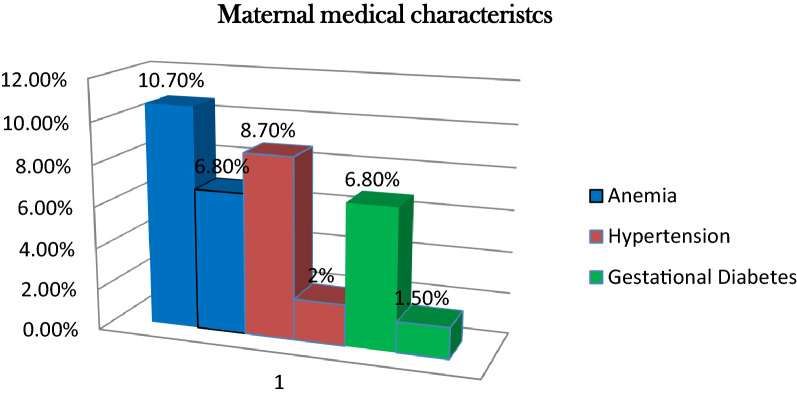
Medical characteristics of mothers who delivered at Ilu Aba Bor, in Public Hospitals, Ethiopia, 2021. Anemia: maternal hemoglobin level <11mg/dl, both pregnancy induced and chronic hypertensive.

Eleven (10.7%) mothers of the cases and fourteen (6.8%) controls had a history of anemia during pregnancy **(**Fig. 1).

### Determinants of birth asphyxia

Hypertension during pregnancy, duration of labor, prolonged rupture of membrane, fetal presentation, parity, gestational age at birth and newborn weight fulfilled the criteria and were potential candidates for the multiple logistic analyses.

By multi-variable logistic regression analysis, duration of labor, fetal presentation, gestational age, and weight of newborn were significantly associated with birth asphyxia.

Newborns of mothers who had prolonged labour were 4.12 times more likely to develop birth asphyxia as compared to those with normal labour (AOR 4.12; [95% CI 1.78, 9.49]. Babies born with non-cephalic presentation were 4.35 times more likely to develop birth asphyxia than their counterparts [AOR 4.35; 95% CI 1.77, 10.67]. This study also revealed that newborns with low gestational age were 5 times more likely to develop birth asphyxia than their counterparts [AOR 5.77; 95% CI 2.62, 12.69]. Low birth weight neonates were 4.43 times higher in developing birth asphyxia than normal ones [AOR 4.43; 95% CI 1.94, 10.13] (Table 4).

**Table 4:** Bivariate & multi-variable logistic regression analysis of birth asphyxia at Ilu Aba Bor zone, in public hospitals, 2021.

Variable	Category	Cases n = 103(%)	Controls n = 205(%)	COR (95% CI)	AOR (95% CI)	*P*-value
Maternal educational status	Not educated	29 (28.2%)	34 (16.6%)	1.97 (1.32, 3.67)	2.44 (1.37, 4.34)	0.006
	Educated	74 (71.8%)	171 (83.4%)	1	1	
Age in years	18-30yrs	58 (56.3%)	87 (42.4%)	1	1	
	< 18 yrs	18 (17.5%)	38 (18.5%)	0.71 (0.37, 1.37)	.93 (.40, 2.13)	0.870
	≥ 30yrs	27 (26.2%)	80 (39.1%)	0.51 (0.41, 0.88)	.65 (.27, 1.18)	0.252
# of ANC	≥ 4	84 (81.6%)	173 (84.4%)	1	1	
Follow up	< 4	19 (18.4%)	32 (15.6%)	1.22 (.65, 2.28)	2.30 (1.17, 4.53)	0.016
Duration of labour	Normal	83 (51.5%)	189 (93.7%)	1	1	
	Prolonged	20 (48.5%)	13 (6.3%)	3.50 (0.13, 0.59)	4.12 (1.79, 9.49)	0.001*
Fetal presentation	Non- cephalic	17 (16.5%)	11 (4.5%)	3.49 (1.57, 7.75)	4.35 (1.77, 10.67)	0.001*
	Cephalic	86 (83.5%)	194 (94.6%)	1	1	
Mode of delivery	SVD	56 (54.4%)	166 (80.0%)	1	1	
	CS	17 (16.5%)	11 (4.5%)	4.58 (2.04, 10.00)	1.79 (.87, 4.69)	0.232
	Instrument	30 (29.1%)	28 (13.7%)	3.18 (1.75, 5.88)	1.47 (.62, 3.45)	0.383
Prolonged rupture of membrane	Yes	10 (9.7%)	11 (5.4%)	1.9 (0.78, 4.62)	1.02 (0.28, 3.70)	0.970
	No	93 (90.3%	194 (94.6%)	1	1	
Parity	Multipara	28 (27.2%)	91 (44.4%)	1	1	
	Primipara	75 (72.8%)	114 (55.6%)	2.14 (1.28, 3.58)	2.14 (1.20, 3.83)	0.010
Hypertension	Yes	9 (8.7%)	4 (2.0%)	4.81 (1.16, 14.8)	3.13 (0.69, 14.17)	0.140
	No	94 (91.3%)	201 (98.0%)	1	1	
Gestational age at delivery	≤ 37 weeks	26 (20.4%)	15 (40.0%)	4.07 (2.15, 8.51)	5.77 (2.62, 12.69)	0.000**
	> 37 weeks	77 (79.6%)	181 (60.0%)	1	1	
Newborn weight in gram	≤ 2500 g	21 (20.4%)	14 (6.8%)	3.49 (1.69, 7.21)	4.43 (1.94, 10.13)	0.000**
	> 2500 g	82 (79.6%)	191 (93.2%)	1	1	

After Bonferroni correction, the threshold of significance was at *p*-value < 0.0038 (*p* = 0.05/13).

Significant values are in bold.

COR, Crude Odds Ratio; AOR, Adjusted Odds Ratio; CI, Confidence Interval; 1, reference category; **p-value < 0.001.

## Discussion

In the current study, the educational status of mothers was not associated with birth asphyxia. In contrast to this finding, studies conducted in Aksum^23^, which revealed that newborns of mothers who were not educated were more likely to develop birth asphyxia compared to those who were educated; Pakistan^29^, Kenya^36^, Nepal^37^, Sweden^24^, and Cameroon^20^, where newborns who were born from mothers with no formal education had a higher risk of birth asphyxia. This might be due to socio-demographic characteristics of study participants, sample size difference, observer and measurement bias, and also an influence on caregiving behavior during pregnancy. However, this study is supported by the studies conducted in Gondar^12^ and Malawi^38^ which reported that educational status was not associated with birth asphyxia. Although maternal education status was not associated with birth asphyxia, it was noticed that maternal education increases, the prevalence of asphyxia.

Maternal age at delivery was not significant for the birth asphyxia in this study. This might be due to the fact that pregnancy and labor in all age groups cannot predict the occurrence of birth asphyxiation if they are effectively managed. Our study finding is consistent with the study findings in Pakistan^25^, Kenya^26^ Thailand^39^, and Gondar^12^ which reported that maternal age was not the determinant factor for birth asphyxia. Moreover, the study in Iraq^30^ revealed that estimates of young (˂18 years) mothers were not associated with asphyxiation. However, our findings are inconsistent with a study conducted in Kenya^36^ that reported that the mother's age was a significant risk factor for developing birth asphyxia. This contrast might be due to the different sample sizes or the type of setting in which the study was conducted.

In a current study, the amount of antenatal care follow up was not significantly associated with birth asphyxia. This finding is congruent with a study done in Rwanda^21^; evidence showed that those who had not had full antenatal follow-up were associated with developing birth asphyxia. The possible explanation was that the majority of women may not be expected to be familiar with or appropriate preventive attitudes with respect to birth asphyxia during their pregnancies (21).

In this study, neonates of mothers who had prolonged labor were 4.12 times more likely to develop birth asphyxia than those with normal labor. This finding is similar to studies done in Aksum^23^, Gondar^12^, Pakistan^25^, and Rwanda^21^, which revealed that the likelihood of developing birth asphyxia was higher in newborns born to mothers with a longer labor time. Moreover, the study from Kenya^36^ also indicated that duration labour was another important risk factor of asphyxia. This may be because the mother's pelvis is not suitable for the newborn's head or does not have sufficient contraction, or the cervix is obliterated and the newborn is large^37^. It is also clear that if there is prolonged labor, there is a high chance that the fetus will be afflicted and result in birth asphyxia. In addition, studies in Nigeria^31^ also showed that prolonged labor is associated with fetal and maternal exhaustion and also with fetal distress, which results in birth asphyxia.

Furthermore, this study found that neonates born with non-cephalic presentation were 4.35 times more likely to develop birth asphyxia than those with cephalic presentation. This finding was similar to studies done in Pakistan^40^ and Iraq^41^, which revealed that fetal malpresentation was significantly associated with the occurrence of asphyxiation. This might be due to the fact that the fetus experiences oxygen deprivation by changing fetal heart rate, fetal movements, and increasing meconium in the amniotic fluid. In addition, malpresentation of the fetus increases the risk of numerous complications, such as umbilical cord prolapse/compression, which can lead to severe suffocation^36^.

Contrary to a study conducted in Indonesia^42^, which indicated the prolonged rupture of membranes (> 18 h) was the most significant risk factor for birth asphyxia, This contradiction might be due to the sample size difference, since the study in Indonesia was a small sample size (70 participants), the setting difference, and the compared participants between the two studies.

This study revealed that the probability of developing birth asphyxia was not associated with parity. This finding is contrasting to the studies done in Aksum^23^, Pakistan^29^, Nigeria^27^ and Kenya^36^. This discrepancy might be due to hawthorn effect, method difference, and possible cofounder example study in Pakistan. Besides, this finding is inline with the study done in Dessie hospital^43^ reported that parity had no significant association with birth asphyxia.

The current study showed that newborns weighing less than 2.5 kg were 4.43 times more likely to develop birth asphyxia than newborns delivered with a weight greater than 2.5 kg. This result is similar to that of the studies done in Aksum^20^, Gondar^12^, Rwanda^20^, Pakistan^25^ and Kenya^36^. This might be due to low birth weight newborns usually having immaturity of the lungs and impaired respiratory muscles^44^.

Finally, in the current study, newborns with low gestational age were 5.77 times more likely to develop birth asphyxia than their counterparts. Our finding is consistent with the study findings in Pakistan^25^ and Kenya^36^. This may be due to the fact that premature babies face multiple medical conditions, including organ system immaturity, especially lung immaturity, which leads to respiratory failure that causes birth asphyxia^30^. However, the studies in Gondar^12^ and Dessie hospital^43^ evidenced that the age of newborns had no significant association with birth asphyxia. The discrepancy may also be due to the decision of *p*-value (*p* ≤ 0.2) which is different from the study (*p* < 0.25) which might result in losing the important variable, and measurement.

## Strength and limitations of the study

The cases and controls were taken from the same setting except for the outcome difference, the assessment of model fitness using different measures, and the reliability test of the questionnaire. Referral bias was avoided by pooling the results across both referral and general hospitals together, since the study was of an institutional-based nature, it cannot represent the whole community.

## Conclusion

Predictors were maternal and newborn-related characteristics. Though these predictors were mainly related to prolonged duration of labour, non-cephalic fetal presentation, premature infants and low birth weights were the independent predictors of birth asphyxia. So, improving the utilization of parthograph during labour, dedicating to giving the correct, quick, and accurate diagnosis and proper management of pathological disorders during pregnancy and delivery, more attention to newborns of low birth weights and preterm newborns at birth by providing a good environment for childbirth, can reduce severe birth asphyxia.

## Data Availability

The data set used and/or analyzed during the current study available from the corresponding author on reasonable request.

## Abbreviations

ANC: Antenatal Care
AOR: Adjusted odd ratio
APH: Antepartum hemorrhage
BA: Birth asphyxia
CI: Confidence interval
COR: Crude odd ratio
CP: Cerebral palsy
CS: Caesarean section
CSA: Central Statistical Agency
FMOH: Federal Ministry of Health
HIE: Hypoxic ischemic encephalopathy
HIV: Human immunodeficiency virus
MDG: Millennium development goals
MNCH: Maternal Neonatal and Child Health
NICU: Neonatal Intensive Care Unit
OR: Odd ratio
RHB: Regional Health Bureaus
SDGs: Sustainable development goals
SVD: Spontaneous vaginal delivery
UNICEF: United Nation Children’s Fund
WHO: World Health Organization
